# Predicting Factors Affecting Adolescent Obesity Using General Bayesian Network and What-If Analysis

**DOI:** 10.3390/ijerph16234684

**Published:** 2019-11-25

**Authors:** Cheong Kim, Francis Joseph Costello, Kun Chang Lee, Yuan Li, Chenyao Li

**Affiliations:** 1 SKK Business School, Sungkyunkwan University, Seoul 03063, Korea; saga@g.skku.edu (C.K.); f.costello@g.skku.edu (F.J.C.); liyuan1108@skku.edu (Y.L.); lichenyao@skku.edu (C.L.); 2 Airport Business Analytics, Economics Department, Airport Council International (ACI) World, 800 rue du Square Victoria, Suite 1810, Montreal, QC H4Z 1G8, Canada; 3 Department of Health Sciences & Technology, Samsung Advanced Institute for Health Sciences & Technology (SAIHST), Sungkyunkwan University, Seoul 06355, Korea; 4 Creativity Science Research Institute (CSRI), Sungkyunkwan University, Seoul 03063, Korea

**Keywords:** public health, health informatics, adolescent obesity, General Bayesian Network, what-if analysis, data mining

## Abstract

With the remarkable improvement in people’s socioeconomic living standards around the world, adolescent obesity has increasingly become an important public health issue that cannot be ignored. Thus, we have implemented its use in an attempt to explore the viability of scenario-based simulations through the use of a data mining approach. In doing so, we wanted to explore the merits of using a General Bayesian Network (GBN) with What-If analysis while exploring how it can be utilized in other areas of public health. We analyzed data from the 2017 Korean Youth Health Behavior Survey conducted directly by the Korea Centers for Disease Control & Prevention, including 19 attributes and 11,206 individual data points. Our simulations found that by manipulating the amount of pocket money-between $60 and $80-coupled with a low-income background, it has a high potential to increase obesity compared with other simulated factors. Additionally, when we manipulated an increase in studying time with a mediocre academic performance, it was found to potentially increase pressure on adolescents, which subsequently led to an increased obesity outcome. Lastly, we found that when we manipulated an increase in a father’s education level while manipulating a decrease in mother’s education level, this had a large effect on the potential adolescent obesity level. Although obesity was the chosen case, this paper acts more as a proof of concept in analyzing public health through GBN and What-If analysis. Therefore, it aims to guide health professionals into potentially expanding their ability to simulate certain outcomes based on predicted changes in certain factors concerning future public health issues.

## 1. Introduction

Due to changes in our lifestyle and eating habits, the world’s obese population has more than doubled from 0.857 billion to 2.1 billion since 1980. Additionally, according to World Health Organization (WHO), it is believed that by 2025, a total of 260 million children worldwide will be overweight, with more than 90 million categorized as obese or worse [1]. Obesity in adolescence is one of the most serious factors causing premature disease and the demise of health in later life [2]. According to the government of Korea, it was reported in 2018 that the rate of obesity among adolescents in South Korea was 10.8%, which means that at least one in every ten adolescents is obese [3].

With this rise in obesity, being able to identify potential causes can help to find preventive measures. Several studies have been conducted in order to explain the factors that cause obesity [4], citing a variety of factors such as: economic factors, such as the cost of eating; state-level prices of grocery food; restaurant prevalence; cigarettes; and alcohol [1]. However, prior research has only considered a few factors within their models, usually implementing simple statistical analysis [4], or using simple classification [5]. In this study, we propose using a more holistic approach whereby classification is integrated with a prediction model that allows the user to adjust certain factors in order to find out what the outcome could be based on this change. Thus, this paper implements the use of a General Bayesian Network (GBN) to identify and classify factors and then uses What-If analysis to then build scenarios on obesity. The use of such a tool is not only useful for obesity research but can also be directly transferred to other areas of health informatics in a way to predict future health outcomes.

Prior research has focused on the use of a GBN in health informatics [6], including its use with a Markov Blanket (MB) to understand obesity [7]. Although this prior research has shed light on obesity within health informatics, it has the following shortcomings. First, is the use of generally accepted influencing factors, such as dietary structure, physical exercise, cigarettes, and alcohol to help predict obesity. Also, although there are many factors that could be involved in the development and maintenance of obesity among a population, there are too many to be investigated in this proof of concept study. Therefore, this study’s focus was more on parameters that are less obvious, such as education and wealth of the family, smartphone usage, pocket money, time spent studying, academic performance, and parents’ education level. These factors have more profound and practical significance in the context of reality. Additionally, we take the analysis a step further compared with this prior work by implementing a What-If analysis based upon the results from the GBN.

### 1.1. Factors Affecting Adolescent Obesity

Adolescence is a pivotal period of one’s life, in which habits are difficult to shake off once learned. Habits such as eating and exercise are ones that adolescent adults struggle with the most [8]. In particular, unhealthy habits, such as smoking, drinking, lack of exercise, and overeating, are known to be direct causes of chronic illnesses, including obesity [9]. Obesity not only has a negative effect on physical, psychological, and emotional development in growing adolescents, but research shows that one-third of adolescent obesity cases leads to adult obesity in later life [9]. In addition, a higher score of Body Mass Index (BMI) (≥25 kg/$m^{2}$) in adults is known to be deeply related to mortality and the occurrence of cardiovascular disease [10]. In addition, the socioeconomic characteristics of adolescents have been reported to have an effect on one’s health. For example, research has shown that elder elementary school students living within residential areas are at greater risk of obesity [11]. Studies have also focused on an ecological model that suggests that individual, interpersonal, organizational, and community characteristics can interact and affect individual behavior and health [12].

Lee et al. found an association between BMI and a mothers’ education level (low) and a lower age of adolescents (middle school). Also, it was found in urban areas for boys who’s fathers’ education level was low [4]. A lack of exercise, increased sitting time, and changes in dietary habits have also been seen as factors [13]. One of the main drivers of this lifestyle change is the recent prevalence in the use of information communication technologies, such as the Internet and smartphones [14]. Previous work has indicated that adolescents addicted to the Internet were more likely to be overweight [14]. Furthermore, a higher use of devices with screens, including computers, video games, smartphones, and tablets, has been seen to increase obesity risk factors in adolescents [11]. In particular, smartphone use has been shown to be related to obesity-related lifestyles, such as watching TV and bad eating habits. Furthermore, it has been shown that teenagers who have more than two hours use of a smartphone a day have higher BMI scores comparatively [11]. Park and Song found that the risk of obesity is much higher when the main reasons for engaging with a smartphone is for entertainment purposes compared with educational purposes, i.e., studying apps [1]. Conversely, their study found the risk of obesity to be lower when the smartphone is used for social purposes [1]. In addition, there has been found to be a significant moderating effect between the quality of sleep and one’s BMI. Hence, sleep quality should be considered as a factor for the prevention and management of obesity [3].

### 1.2. Data Mining for Predicting Obesity

Predicting obesity is a social challenge and, therefore, researchers have been studying obesity from various perspectives [15,16,17,18,19,20]. Due to its complexity, an increasing number of researchers are turning to the use of data mining and machine learning tools [21]. Bhattarai et al.’s [22] research used text classification to help predict obesity. Zhang, Tjortjis, Zeng, Qiao, Buchan, and Keane’s [5] paper provided a comparison between seven data mining techniques, including Bayesian Networks (BN). Research implementing Artificial Neural Network (ANN), Naïve Bayes Network (NBN), and Decision Tree (DT) have proven to be applicable to predicting childhood obesity [23,24]. However, the prediction of adolescent ages is slightly more complex and thus difficult for many algorithms to handle. This is because the reasons that lead to being overweight or obese are complicated, involving not only physiological but also genetic, sociological, and many psychological factors [25].

### 1.3. General Bayesian Network (GBN) and What-If Analysis

GBN is a directed acyclic graph in which nodes represent domain variables, and arcs between nodes represent probabilistic dependencies [26]. GBN is a popular type of probabilistic network, which has found many different applications in areas such as online learning [27], software development [28], medical informatics [24], social media analytics [29], environmental concerns [30], and in-classification tasks [31]. Finding the probability of an event given a set of prior evidence can be solved through Bayesian nets with sequential applications of Bayes Theorem. It is important to find out the inverse probability, i.e., when we know *P(B|A)*, then we can use a Bayesian network to find out P(A|B). Overfitting often happens in GBN when the parameters of datasets reach the extents that the computer can cope with. Feature selection (FS) can be applied to solve the above problem through the removal of redundant and irrelevant features. For this reason, we implemented the use of the Markov Blanket, which has been used for this feature in previous papers [32,33,34].

(1) $$P\left(A|B\right)=\frac{P\left(B|A\right)P\left(A\right)}{P\left(B\right)}$$

What-If analysis can be described as a data-intensive simulation that inspects the behavior of a complex system under some given hypothesis (called scenarios) [35]. More pragmatically, What-If analysis measures how changes in a set of independent variables impact a set of dependent variables with reference to a given simulation model [36]. What-If analysis can be used to solve several complicated problems, especially for highly complex research models where the number of variables would make it difficult for general classification models to find patterns [37]. Furthermore, if the What-If analysis is integrated with a GBN model, important matters can be easily perceived by researchers through the graphical presentation [38].

What-If analysis recalculates variables using substituted presumption to discover another consequence. What-If analysis can be represented by $x_{i}$ as the *i*th input of *x* = {*x*1, ..., *xn*}, and ($x_{i}$, $x_{j}$) is the sub-vector of $x_{i,j}$, then, the What-If analysis is deduced with a decomposition of the function *η*(∙), and it can be fully written in the following [37]:

(2) $$y=(X)=(Y)+\sum_{i=1}^{d}z_{i}\left(x_{i}\right)+\sum_{i<j}z_{i,j}\left(x_{i,j}\right)+\sum_{i<j<k}z_{i,j,k}\left(x_{i,j,k}\right)+\ldots +z_{1,2,\ldots ,d}\left(x\right)\;\mathrm{where}\;z_{i}\left(x_{i}\right)=E\left(Y|x_{i}\right)-E\left(Y\right),\;z_{i,j}\left(x_{i,j}\right)=E\left(Y|x_{i,j}\right)-z_{i}\left(x_{i}\right)-z_{j}\left(x_{j}\right)-E\left(Y\right),\;z_{i,j,k}\left(x_{i,j,k}\right)=E\left(Y|x_{i,j,k}\right)-z_{i,j}\left(x_{i,j}\right)-z_{i,k}\left(x_{i,k}\right)-z_{j,k}\left(x_{j,k}\right)-z_{i}\left(x_{i}\right)-z_{j}\left(x_{j}\right)-z_{k}\left(x_{k}\right)-E\left(Y\right).$$

In this research, GBN MB with What-If analysis was implemented in obesity to suggest the impact of various parameter changes on the incidence of obesity. Note that the parameters that we have selected for this paper are for the purposes of demonstration, rather than for the purposes of providing an overall medical solution for obesity.

## 2. Methodology

### 2.1. Procedure

First, we compared the GBN classifier with some common algorithms known for high accuracy in solving complex datasets. Next, accuracy, AUC, and F-measure were performed in order to test the performance of the GBN models against the other chosen classification models. Furthermore, in order to provide a practical implication for predicting adolescent obesity from this paper, What-If analysis was simulated using the result from the GBN models derived from the given dataset on obesity. The open source tool WEKA was used to implement classifiers on the dataset and can be found at the following URL: https://www.cs.waikato.ac.nz/ml/weka/.

### 2.2. Dataset

This paper uses the raw data of the 2017 Korean Youth Health Behavior Survey conducted by the Korean Centers for Disease Control & Prevention. The target collection group of the 13th Adolescent Health Form Online Survey (KCDC, 2017) had a participation of 62,276 in April 2016. The online survey of youth health behaviors was based on and approved by the government approval statistics committee (Approval No. 11758) and the National Health Promotion Act (Article 19). The 2017 Korean Youth Health Behavior Survey surveyed 123 questions in 15 areas, including smoking, drinking, and physical activity, and calculated 216 indicators. This paper used the sociological characteristics of the population, health morphological characteristics, smartphone use characteristics, obesity, and other related projects.

### 2.3. Data Pre-Processing

First, we deleted responses that had missing values and also removed the irrelevant attributes that could not help in evaluating obesity. Second, we removed the data for respondents that had no prior smartphone use, as well as data from respondents who answered as “don’t know” to the smartphone usage section. Third, we calculated the degree of obesity by the following steps: (1) the BMI is calculated by dividing the body weight by the square of the height (Kg/$m^{2}$). (2) According to the 2007 growth chart for children and adolescents, as shown in Table 1, four categories are defined. Over the 95th percentile is obesity, the 85th percentile to 95th percentile is overweight, 5th percentile to 85th percentile is normal, and less than 5th percentile is underweight. Fourth, we selected 33 attributes based on three key articles, and the attributes are processed as follows: (1) since the classification of BMI is obtained according to gender and age, and the level of school is directly related to age, we deleted these four attributes: “AGE”, “SEX”, “SCHOOL”, and “GRADE”. (2) We combined some relevant attributes into one attribute, specifically, “F_BR”, “F_FRUIT”, “F_VEG”, and “F_MILK” were transformed into “Healthy_Eating”; “F_FASTFOOD”, “F_INSTND”, “F_CRACK”, “F_SODA”, “F_CAFFINE”, and “F_SWDRINK” were transformed into “Unhealthy_Eating”; “PA_SWD_S” and “PA_SWK_S” were transformed into “Sitting_Time_Study (min)”, and “INT_SP_WD” and “INT_SP_WK” were transformed into “Smartphone_Time (min)”.

After the data-preprocessing was complete, the revised dataset had 4 classes, 19 attributes, and 11,206 data instances (see Appendix A for a full breakdown).

## 3. Results

### 3.1. Performance Comparison of GBN with Other Classifiers

In order to confirm the suitability of our GBN-MB model, we have compared the results of Accuracy, F-measure, and AUC (area under the curve), which are commonly used to evaluate the performance of machine learning techniques [39,40,41], of the model to the other machine learning classifiers. Results indicated that for this specific set of data, the best fit was obtained with the GBN model that embedded the MB. Based on the scores obtained from accuracy, GBN-MB showed an accuracy [39] of 53.703%, an F-measure [40] of 0.535, and an AUC (area under the curve) [41] score of 0.758 (see Table 2). This result proved that the use of MB could enhance the performance of a GBN network’s classification ability. Hence, we recommend GBN-MB to predict adolescent obesity, as its performance is slightly better than all other classifiers. GBN and Bagging (BA) also were found to have comparatively reasonable scores; however, they fell short in all the tests. A Support Vector Machine (SVM) has been considered as a trustful machine learning approach among researchers [42,43,44]. However, surprisingly, for this dataset, Support Vector Machine (SVM) showed very poor results in accuracy (45.431%), together with the Naïve Bayes Network (NBN) (45.627%) compared to the other models implemented. Despite GBN-MB showing the best performance in all the tests, the results suggest that in the domain of adolescent obesity prediction, scores are fairly low, and thus, the complexity of the problem remains clear. However, moving forward, the inclusion of GBN-MB and potentially GBN and Bagging (BA) could be considered.

### 3.2. Chosen Factors for What-If Analysis

In order to find interesting relationships in the obesity data, we attempted to shift the lens in which previous research has viewed obesity. Although there are many common factors that could be used to explain the cause of obesity, this study focused more on unique factors that were not dealt with in previous research. Hence, we chose the following features for further analysis: “Obesity_Level”, “Pocket_Money (KRW)”, “Wealth”, “Pressure”, “Sitting_Time_Study (min)”, “Sleeping_Quality”, “Education_Mother”, “Education_Father”, “Academic_Performance”, “Smartphone_Time (min)”, and “Smartphone_Service”.

Table 3 shows the sensitivity to findings of each factor node with respect to each influencing factor. There would be a higher influence of the variable on the target nodes when the value of mutual information was higher. The same principle can be applied to entropy (%), which means the percentage of each influencing factors on the dependent variable is greater (Obesity_Level).

As Table 3 shows, Pocket_Money (KRW) was found to be the most influential factor on the level of obesity, while Sleeping_Quality, Sitting_Time_Study (min), Academic_Performance, Wealth, and Pressure follow. Through this result, it could be interpreted that the amount of pocket money which adolescents receive every week can affect the somatotype. In addition, the quality of sleeping and the time adolescents spent sitting for study ranked second and third, respectively, indicating that healthy lifestyles are significant to the bodily form during one’s adolescence. The academic performance, family wealth, and pressure level ranked fourth, fifth, and sixth, respectively. All the important influencing factors will be discussed in detail by sensitivity analysis in the following paragraphs.

### 3.3. What-If Analysis

What-If analysis can be used to solve several complicated problems, especially for highly complex research models. For example, to determine the factors that affect adolescent obesity, we needed to manipulate the maximum and minimum output of the models. Therefore, in this study, we have generated 13 scenarios for obesity, as shown in Table 4.

Figure 1, Figure 2, Figure 3, Figure 4, Figure 5, Figure 6, Figure 7 and Figure 8 show the results of the What-If analysis for each scenario. Figure 1 shows the initial state of the Bayesian Network previously obtained in the classification section of this paper, in which the initial value of ‘Obesity’ is 22.8.

**Figure 2:**
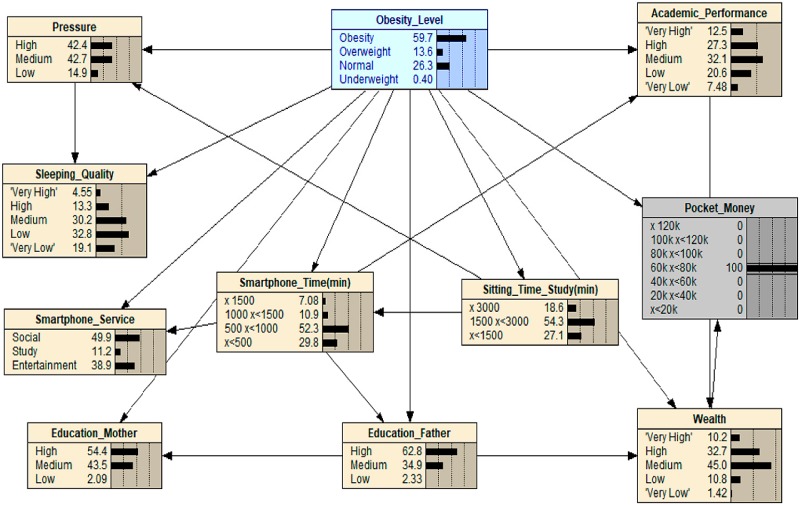
What-If analysis when Scenario 1 is applied: note that the grey variable represents the variable that has been manipulated and, thus, through this, we can try to understand what effect this has on the other relationships in this What-If analysis graph, including the effect it has on the final outcome *obesity*. The graphs that follow on from this one have the same methodological approach.

When scenario 1 is applied (see Figure 2), the medium value of ‘Pocket_Money’ (60 k ≤x<80 k) is applied, and as can be seen, the value of ‘Obesity’ increases from 23.0 to 59.7. This result shows that adolescents with weekly pocket money around 60 k to 80 k Korean Won are more likely to become obese. When the level of obesity was manipulated with maximized or minimized levels of ‘Pocket_Money’, the value of ‘Obesity’ became close to 0. This result indicates that adolescents who have either a large budget or low budget for pocket money have a near to zero chance of becoming obese. Potential reasons for this kind of finding could be inferred as those adolescents who have larger budgets might pay more attention to body management or spend money on bigger ticket items compared with a small ticket item such as fast food. In contrast, if one has a smaller budget, or none at all, they cannot afford to spend money on luxuries such as fast food or snacks. Hence, our results indicate that only those who have a reasonable pocket money budget may face the large risk of being obese.

**Figure 3:**
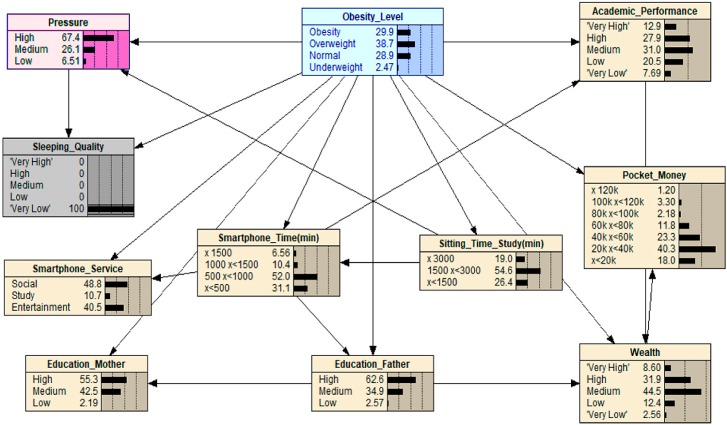
What-If analysis: when scenario 2 is applied.

When scenario 2 is applied (see Figure 3), which sets ‘Sleeping_Quality’ to ‘Very bad’, the value of ‘Obesity’ increases from 22.8 to 29.9. Even though the change is not so dramatic, we can still find that adolescents with poor sleeping quality are more likely to be obese. At the same time, we observed that the value of ‘Pressure’ increased significantly from the initial 39.1 to 67.4, indicating that high pressure brings about bad sleeping quality. This result presumes that adolescents may manage pressure from studying and exams by eating, leading to a higher probable chance of obesity. Essentially, eating could be seen as a coping mechanism to deal with times of pressure.

**Figure 4:**
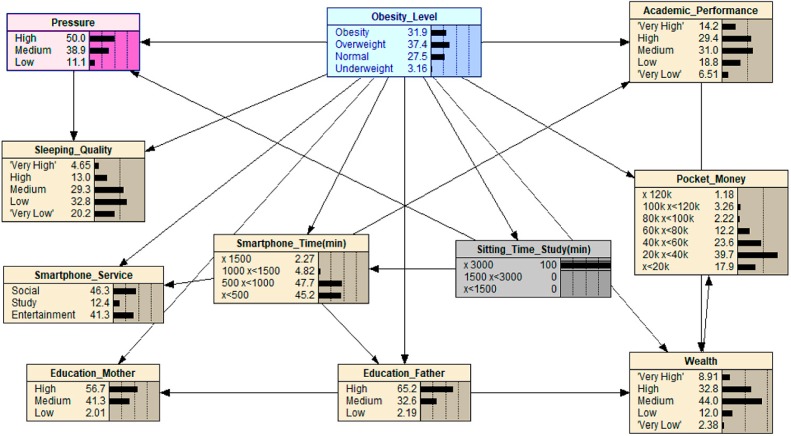
What-If analysis: when scenario 3 is applied.

When we apply scenario 3 (see Figure 4), time spent studying is set to the maximum value. This has the effect of slightly reducing smartphone usage, but more obvious is the amount of pressure on adolescents. Pressure in a state of high time spent studying increases in the high category from 39.1 to 50.0, having a knock-on effect in terms of obesity levels (22.8 > 31.0). Therefore, scenario 3 confirms what is generally deemed as common knowledge when it comes to excessive studying.

**Figure 5:**
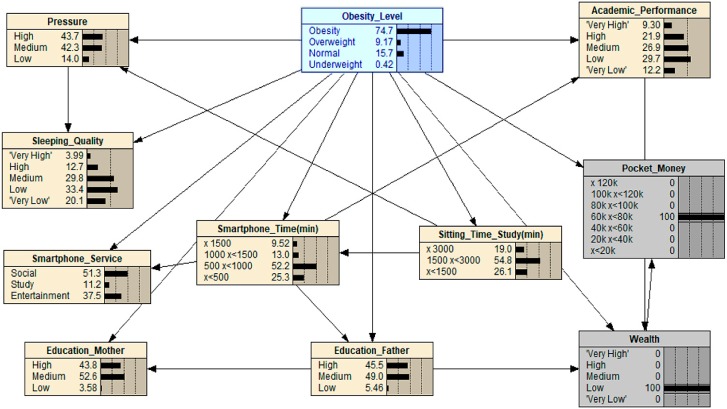
What-If analysis: when scenario 4 is applied.

For scenario 4, we selected a medium budget for ‘Pocket_Money’ and also set the ‘Wealth’ to ‘Low’ (see Figure 5). As can be seen in the results, the value of ‘Obesity’ has increased from 22.8 to 74.7, indicating that the family economic condition compounded with the amount of money that adolescents received as pocket money can influence the adolescents’ obesity level. This finding reflects the fact that families who are on the lower end of the economic spectrum have to buy cheaper fast and processed foods. This then has a knock-on effect on the fact that this choice from parents then reflects into the adolescent’s choice in how they spend their pocket money, i.e., further fast food, or unhealthy snacks.

**Figure 6:**
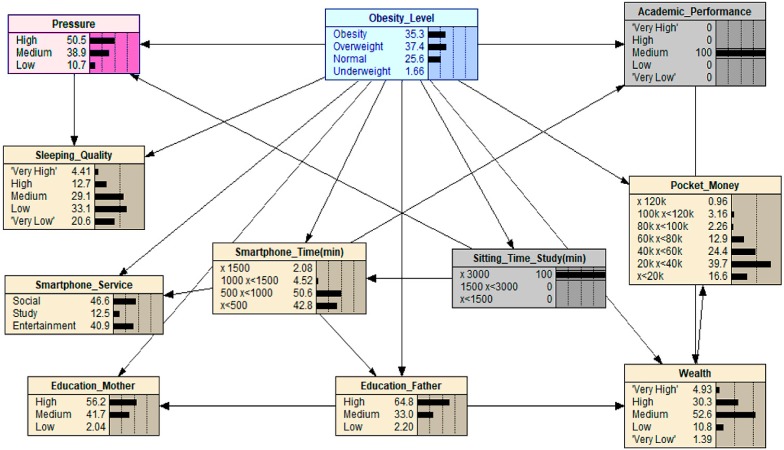
What-If analysis: when scenario 5 is applied.

In Figure 6, we applied scenario 5, which maximizes the level of ‘Sitting_Time_Study’, and also implemented a middle value for ‘Academic_Performance’. The result showed that the value of obesity increased from 22.8 to 35.3. Upon testing the significance of this feature while maximizing or minimizing the level of ‘Academic_Performance’, we found no significant change to obesity. In addition, the change in obesity level is not significant when we maximize or minimize the level of ‘Academic_Performance’ in conjunction with maximized ‘Sitting_Time_Study’. We can reasonably suggest that the students that are in the medium percentile of intelligence might have to put more effort into their studying efforts, which ultimately leads to the increase of time spent sitting down studying. Therefore, the value of obesity also increased, respectively. At the same time, it is obvious that the value of high ‘Pressure’ also increased from 39.1 to 50.5, a greater increase compared with scenario 3. This suggests that those who spend more time studying who fall within the medium percentile of intelligence suffer from a higher pressure from studying.

**Figure 7:**
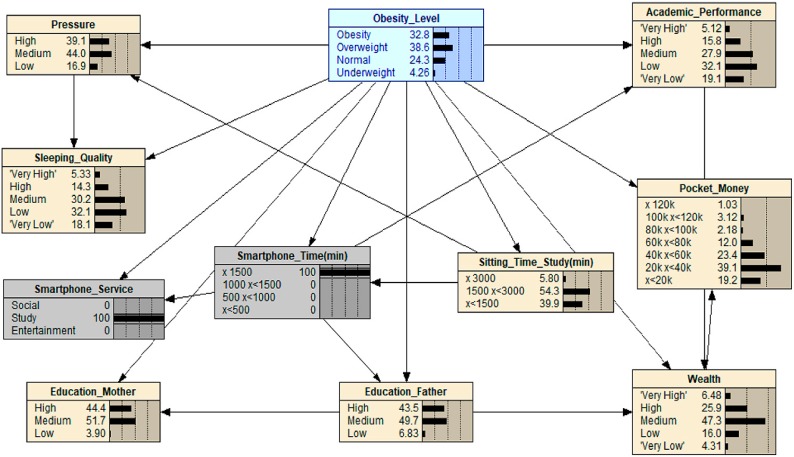
What-If analysis: when scenario 6 is applied.

By maximizing the level of ‘Smartphone_Time’ and setting the ‘Smartphone_Service’ configuration to “study”, we applied scenario 6 to the obesity dataset (see Figure 7). The results from this adjustment show how obesity went from 23.0 to 32.8 in probability. This scenario shows similar aspects to the results that were seen in scenario 4, albeit with different manipulation of the variables. As can be seen in Figure 7, the increase in studying time spent on a smartphone results in an increase in obesity level. As smartphones continue to be an integral part of adolescent life and grow as a source of entertainment, studying, and socializing for these young people, it is leading to a decrease in physical activity and thus, can help to explain why obesity will increase, even if the technology is used for studying.

**Figure 8:**
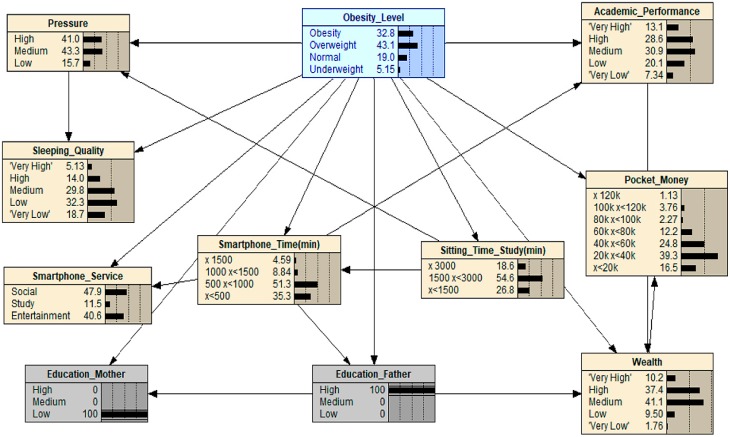
What-If analysis: when scenario 7 is applied.

In our final scenario, we wanted to investigate the relationship between adolescent obesity and their parents’ education level. To the best of our knowledge, this has not been explored in previous research and, thus, could shed light on an unknown area within the obesity debate. To achieve this supposition, we adjusted the value of the father’s education data point to the maximum value as well as that of the of the mother’s education data point to the minimum value to see if the educational backgrounds of parents in general could influence the obesity levels of their children, as shown in scenario 7, (see Figure 8). In doing this, we are not saying that the people questioned in this survey will change their education; instead, this allows a general overview of what potentially would happen if this trend was to continue and become more extreme. The result showed that obesity levels were likely to increase from 23.0 to 32.8. This combination proved to be the highest change in obesity levels when compared to other scenarios such as “Maximized mother and father education” and “Minimized mother and father education”. A plausible explanation of this is that the high level of a father’s education results in higher level job, one that has more demanding hours and responsibility. Consequently, this takes time away from the father’s family life and leaves the mother to rear the children in a more independent household. Coupling this with a low education level for the mother creates the potential for the mother to not correctly nourish the children with correct amounts of food or nutrition and can lead to the mother making poor choices when it comes to eating out. All these factors ultimately lead to a greater risk of adolescent obesity.

## 4. Discussion

With the remarkable improvement in people’s socioeconomic living standards, the obesity of adolescents has become a serious social problem in the developed world. Over the past 30 years, the rate of adolescent obesity has quadrupled, increasing health care and their associated costs, as well as increasing the risk of disease. Although obesity rates have been halting in countries like the United States [45], more recently, in developed countries, such as South Korea, obesity in adolescents is dramatically increasing. With this, the associated risks and health concerns, for example, increased chance of cancer, are now becoming a serious problem in these countries too [46]. Up until now, much research on obesity and adolescence has been based on case studies, and the analysis of data with common techniques has helped to make people more aware of the issue and increase the level of understanding in this area [47,48]; however, little research has tried to build scenarios that can help to predict the levels of obesity based on computing techniques [6]. The lack of research within this area is great, so this study attempted to fill this gap by applying computing techniques to a public health dataset obtained from the South Korean government in order to try and predict the main contributing factors of adolescent obesity.

This paper focused on predicting the influencing factors of adolescent obesity by using a GBN with an embedded MB. Chung et al. (2013) made recommendations on the use of a GBN within health informatics, and previous research done on data from the UK showed the power of using a GBN for understanding obesity [7]. The results of our study also found a GBN with an MB to be the best classifier for the causes of obesity. However, the classification of causes cannot tell us much, and therefore, we took the focus one step further by adding the power of a What-If analysis. In doing so, and to the best of our knowledge, this paper is one of the first to implement this computing technique on obesity prediction within adolescents. Implications of the results from this analysis are presented next.

### 4.1. Implications

Based on the seven scenarios poised in the What-If analyses, we can reach the following implications. First, in scenario one, adolescents with a reasonable pocket money budget were seen to have a higher chance of obesity. This is also cited in prior research, which suggested that children in China who had more pocket money would more likely consume sugary drinks and eat fast food [49]. Although this study focused on children, continuing a trend from childhood through to adolescence is very possible. However, it is clear that when children turn to adolescence, it is possible that those with considerably larger budgets are more likely to take an interest in activities such as going to the cinema or driving. Thus, money spent on food at childhood may turn into bigger ticket items. In Li et al.’s study [49], they found that the mother’s perception of their caregiving role, plus concern over their child’s health, played an influence on how much money they gave for pocket money. This tie is nicely seen within scenario 7.1. whereby low education of the mother resulted in greater obesity. However, interestingly, when we applied a low economic family scenario alongside a medium level of pocket money, obesity increased dramatically. Summarizing these findings would lead one to imply that low education of the mother (i.e., unawareness of child/adolescents’ health) compounded with a reasonable amount of pocket money is also tied in with a low-income home. Low-income households usually do not have the luxury of affording fresh fruit and vegetables, and thus, fast food and packaged foods are more likely on the menu at home. Thus, poor teaching on behalf of the mother (based on lack of education and funds) leads to unhealthy choices when the child/adolescence has enough pocket money to buy food/snacks.

From scenario two, it shows that as poor quality of sleep increases the chance of being obese, agreeing with prior research that also links body aches with these two factors [50]. Another key outcome seen was with the increase in pressure within adolescents, a factor that increased in quite significantly when sleep deprivation and obesity also increased. This, therefore, adds a psychological element to the obesity epidemic and has been linked in prior research [51]. Lack of sleep, especially in countries like South Korea, is directly linked to higher hours spent studying; however, no link between hours spent studying and obesity has previously been found [52]. This study, however, did find a direct link between increased study time and obesity. Excessive studying can lead to boredom as well as tiredness. From this result, it could be implied that adolescents within these states would then turn to eating in order to eliminate boredom or turn to energy drinks or coffee to relieve fatigue. In both cases, the risk of choosing high sugar snacks and drinks is high. Additionally, excessive studying leads to sleepless nights, as well as late consumption of sugary and caffeinated drinks, which helps to prevent a good night’s sleep. Therefore, our results comply with common knowledge when it comes to the risks of obesity in these particular aspects.

Finally, smartphone use was implied to increase the chances of obesity, when used for studying purposes in scenario six. Although smartphone use has been linked to obesity in prior work [53], it has not specified the usage of the smartphone. This result could imply that when Korean adolescents study, they use their phones as a tool, and therefore, with this, it removes time away from, for example, physical activity. However, contrary to common belief, this result cannot ignore the fact that phones used for entertainment and social purposes may also have a contributing effect on the potential levels of obesity.

### 4.2. Limitations and Future Recommendations

Korea is still a society largely driven by male dominance and a mother and father household. Therefore, some of the scenarios might be unique whereby men are the so-called ‘breadwinners’, and women are expected to be ‘stay-at-home’ mothers. Although some of the scenarios shed potentially new implications on the causes of obesity, the Korean culture may be playing a more dominant factor in these results. This implies that these results may not cross boundaries into countries whereby women play a more liberal or dominant role in the workplace and whereby women are the ‘breadwinners’, and it is instead, the father who stays at home. Furthermore, this result ignores the fact that there is growing evidence that divorced households, i.e., single mothers or fathers, have been shown to have an effect on obesity in preadolescent children [54], and it is very plausible that this effect could spill over into adolescents too.

Another limitation of this study is that we did not consider the effects of factors such as posttraumatic stress disorders, lack of exercise, and a direct reference to diet choices within our scenario building. Recent research has started to make direct links between these factors and obesity [55], and therefore, future research will need to look to include such factors in order to gain a greater insight into the causes of adolescent obesity. Another factor that is sometimes ignored within the public health sphere when it comes to preventing obesity is in the government’s role to potentially limit the number of, or heavily tax fast food restaurants, such as McDonalds and KFC, which are now rapidly growing in Asia [56]. Therefore, future research could also look into the geographical locations of fast food restaurants and the perceived choice adolescents have when it comes to selecting a restaurant.

Because our GBN-MB research model is based on cross sectional data, the relationships are not causal, while they are more associative. In addition, it is difficult to represent the characteristics of all situations within the globe as the dataset used was limited to the case of Korea. Thus, future research should look to study a selection of various data samples from several nations who are suffering from this rise in obesity. This would help to find more meaningful results that have not been derived in this study and could potentially contribute even more by providing a more fruitful solution from the perspective of public health concerns on obesity. In line with this, another limitation is in the ability to translate informatic-based models into real-world health informatics. Therefore, in order to examine the findings within this paper, we suggest that future research should look to employ longitudinal epidemiological data to help support the findings from this paper.

## Figures and Tables

**Figure 1:**
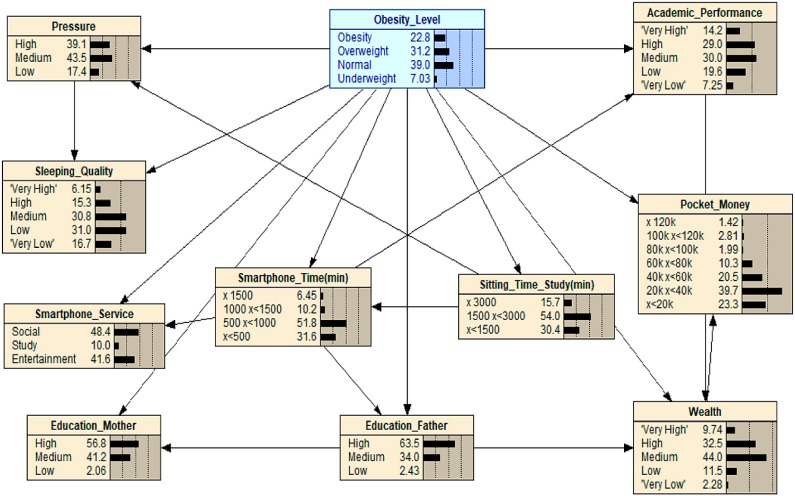
What-If analysis: this is the initial state of the predicted data we extracted from the GBN analysis. From this canvas, we are able to manipulate certain parameters to find out what effect this has on the relationship with other nodes, especially with the dependent variable node obesity.

**Table 1:** Growth chart for children and adolescents in 2007.

Age	Gender	BMI Percentile		
		5th	85th	95th
12	Boy	15.35	23.32	26.35
	Girl	15.20	22.22	24.77
13	Boy	15.82	23.93	27.02
	Girl	15.71	22.83	25.38
14	Boy	16.32	24.40	27.48
	Girl	16.25	23.31	25.83
15	Boy	16.83	24.74	27.77
	Girl	16.78	23.67	26.11
16	Boy	17.33	24.95	27.89
	Girl	17.27	23.89	26.24
17	Boy	17.80	25.08	27.89
	Girl	17.68	23.99	26.24
18	Boy	18.20	25.18	27.85
	Girl	17.96	23.98	26.15

**Table 2:** Performance evaluation of classifiers.

	Accuracy	F-Measure	AUC
GBN-MB	**53.703%**	**0.535**	**0.758**
GBN	52.579%	0.523	0.743
LR	46.689%	0.436	0.688
DT	46.779%	0.467	0.632
SVM	45.431%	0.134	0.661
NN	50.535%	0.495	0.723
NB	45.627%	0.451	0.674
BA	52.588%	0.521	0.742
RSS	51.776%	0.489	0.736
RF	52.570%	0.518	0.743

**Table 3:** Sensitivity of ‘Obesity_Level’ to findings at other nodes.

Nodes	Mutual Information	Entropy (%)	Variance of Beliefs
Obesity_Level	1.80976	100	0.4895308
Pocket_Money (KRW)	0.22196	12.3	0.0246610
Sleeping_Quality	0.03398	1.89	0.0025873
Sitting_Time_Study (min)	0.02494	1.38	0.0027259
Academic_Performance	0.02423	1.35	0.0014365
Wealth	0.02413	1.34	0.0017131
Pressure	0.01420	0.766	0.0010950
Education_Mother	0.00766	0.425	0.0007854
Smartphone_Service	0.00645	0.357	0.0006391
Smartphone_Time (min)	0.00478	0.283	0.0001776
Education_Father	0.00396	0.221	0.0003561

**Table 4:** What-If analysis scenario of factors affecting adolescent obesity.

What-If Analysis Scenario	Obesity Result (%)	
Scenario 1	Select the middle level of Pocket_Money	22.8 → 59.7
Scenario 2	Set Sleeping_Quality to ‘Very low’	22.8 → 29.9
Scenario 3	Maximize Sitting_Time_Study	22.8 → 31.9
Scenario 4	Select the middle level of Pocket_Money and set Wealth to “Low”	22.8 → 74.7
Scenario 5	Maximize Sitting_Time_Study and select the middle level of Academic_Performance	22.8 → 35.3
Scenario 6	Maximize Smartphone _Time & set Smartphone_Service to “Study”	22.8 → 32.8
Scenario 7	Maximize Education_Father and minimize Education_Mother	22.8 → 32.8

Note: obesity result (%) stands for the probability of causing obesity that was calculated by our General Bayesian Network (GBN) models.

## Appendix A

**Table A1:** All attributes included within this study.

Revised Attributes	Original Attributes	Type	Description	Source
Region	CTYPE	Nominal	City type (County, Big city, Small city)	Lee, Kang, Kim, Son, Lee and Ham [4] Park and Song [1] Jang, Oh, Kim and Shin [3]
Academic_Performance	E_S_RCRD		Academic performance	
Pressure	M_STR		How often do you feel under pressure?	
Suicide_Thought	M_SUI_CON		In the last 12 months, have you ever had suicide thought?	
Sleeping_Quality	M_SLP_EN		In the last 7 days, do you think that sleeping time is sufficient?	
Drinking	DRINKING		Have you ever had a drink?	
Smoking	SMOKING		Have you ever smoked cigarettes?	
Education_Father	E_EDU_F		Father’s educational level	Lee, Kang, Kim, Son, Lee and Ham [4]
Education_Mother	E_EDU_M		Mother’s educational level	
Wealth	E_SES		Family economic level	Jang, Oh, Kim and Shin [3]
Pocket_Money (KRW)	E_ALLWN		Average pocket money per week	
Healthy_Eating	F_BR	Numeric	During the last 7 days, how many days did you eat breakfast?	Lee, Kang, Kim, Son, Lee and Ham [4] Park and Song [1] Jang, Oh, Kim and Shin [3]
	F_FRUIT		During the last 7 days, how often did you eat fruit?	
	F_VEG		During the last 7 days, how often did you eat vegetables?	
	F_MILK		During the last 7 days, how often did you drink milk?	
Unhealthy_Eating	F_FASTFOOD		During the last 7 days, how often did you eat fast food?	
	F_INSTND		During the last 7 days, how often did you eat instant noodles?	
	F_CRACK		During the last 7 days, how often did you eat snake?	
	F_SODA		During the last 7 days, how often did you drink carbonated drinks?	
	F_CAFFEINE		During the last 7 days, how often did you drink coffee or energy drinks?	
	F_SWDRINK		During the last 7 days, how often did you drink sweet drinks?	
Exercise_60 min	PA_TOT		In the past 7 days, the number of days that take more than 60 min of exercise.	Lee, Kang, Kim, Son, Lee and Ham [4] Park and Song [1] Jang, Oh, Kim and Shin [3]
Exercise_20 min	PA_VIG		In the past 7 days, the number of days that take more than 20 min of strenuous exercise.	
Sitting_Time_Study (min)	PA_SWD_S		In the last 7 days, how many hours do you spend sitting for study per day on weekday?	Lee, Kang, Kim, Son, Lee and Ham [4]
	PA_SWK_S		In the last 7 days, how many hours do you spend sitting for study per day on weekend?	
Smartphone_Time (min)	INT_SP_WD		In the last 7 days, how many hours do you spend using smartphone per day on weekday?	Park and Song [1]
	INT_SP_WK		In the last 7 days, how many hours do you spend using smartphone per day on weekend?	
Smartphone_Service	INT_SP_ITEM	Nominal	During the last 30 days, which kind of smartphone service is mainly used?	
Obesity_Level	Obesity		>=95th BMI Percentile	Lee, Kang, Kim, Son, Lee and Ham [4] Park and Song [1]
	Overweight		85th~95th BMI Percentile	
	Normal		5th~85th BMI Percentile	
	Underweight		<=5th BMI Percentile	
